# Green Synthesis of Silver Nanoparticles Using *Acacia ehrenbergiana* Plant Cortex Extract for Efficient Removal of Rhodamine B Cationic Dye from Wastewater and the Evaluation of Antimicrobial Activity

**DOI:** 10.1021/acsomega.3c01292

**Published:** 2023-05-15

**Authors:** Waleed M. Alamier, Mohammed D Y Oteef, Ayyob M. Bakry, Nazim Hasan, Khatib Sayeed Ismail, Fathi S. Awad

**Affiliations:** † Department of Chemistry, Faculty of Science, Jazan University, Jazan 45142, Saudi Arabia; ‡ Department of Biology, Faculty of Science, Jazan University, Jazan 45142, Saudi Arabia; § Chemistry Department, Faculty of Science, Mansoura University, Mansoura 35516, Egypt

## Abstract

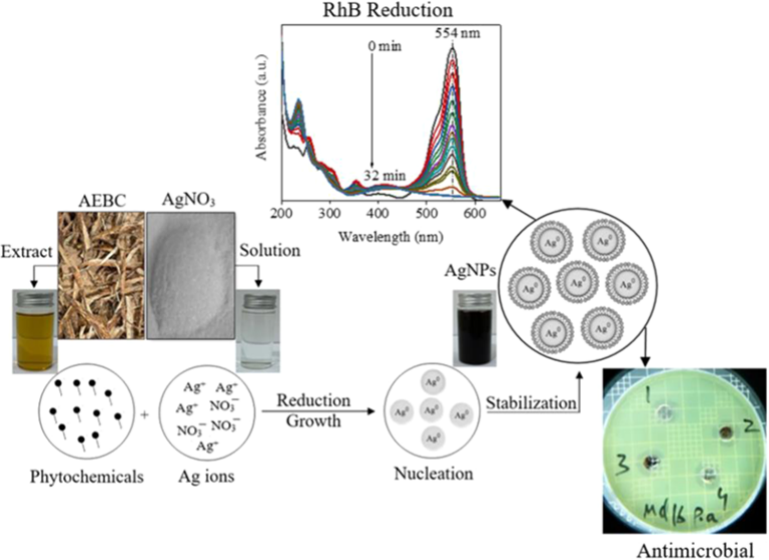

Silver nanoparticles (Ag-NPs) exhibit vast potential in numerous applications, such as wastewater treatment and catalysis. In this study, we report the green synthesis of Ag-NPs using *Acacia ehrenbergiana* plant cortex extract to reduce cationic Rhodamine B (RhB) dye and for antibacterial and antifungal applications. The green synthesis of Ag-NPs involves three main phases: activation, growth, and termination. The shape and morphologies of the prepared Ag-NPs were studied through different analytical techniques. The results confirmed the successful preparation of Ag-NPs with a particle size distribution ranging from 1 to 40 nm. The Ag-NPs were used as a heterogeneous catalyst to reduce RhB dye from aqueous solutions in the presence of sodium borohydride (NaBH_4_). The results showed that 96% of catalytic reduction can be accomplished within 32 min using 20 μL of 0.05% Ag-NPs aqueous suspension in 100 μL of 1 mM RhB solution, 2 mL of deionized water, and 1 mL of 10 mM NaBH_4_ solution. The results followed a zero-order chemical kinetic (*R*^2^ = 0.98) with reaction rate constant *k* as 0.059 mol L^–1^ s^–1^. Furthermore, the Ag-NPs were used as antibacterial and antifungal agents against 16 Gram-positive and Gram-negative bacteria as well as 1 fungus. The green synthesis of Ag-NPs is environmentally friendly and inexpensive, as well as yields highly stabilized nanoparticles by phytochemicals. The substantial results of catalytic reductions and antimicrobial activity reflect the novelty of the prepared Ag-NPs. These nanoparticles entrench the dye and effectively remove the microorganisms from polluted water.

## 1 Introduction

In recent years, the high demand for modern technologies has led to water contamination by various pollutants, including toxic organic dyes and microorganisms. Organic dyes are classified into three types: cellulose fiber dyes (direct, sulfur, and indigo dyes), protein fiber dyes (azo, anthraquinone, triarylmethane, and phthalocyanine dyes), and synthetic fiber dyes (disperse and basic dyes).^1−7^ These dyes are used in various industrial, medical, and biological applications, such as printing, staining, food, textile, paper, drug production, and painting.^8−10^ However, releasing the aqueous industrial waste of these toxic dyes into natural water resources without pretreatment poses a significant threat to the environment and ecosystem. Such contamination can alter the properties of the soil, which threatens the fauna and flora in the vicinity, in addition to causing the death of microorganisms. Moreover, the presence of toxic dyes above the drinking water limit can cause several chronic and severe diseases, including skin problems, eye irritation, lung disease, kidney and liver damage, dyspnea, cancer, and ulcers.^11,12^ Additionally, the presence of microorganisms such as bacteria and fungi in natural water resources can cause many water-borne diseases, such as cholera, typhoid, giardia, dysentery, *Candida albicans*, microsporidiosis, and *Candida parasitosis*.^13−15^ Therefore, it is mandatory to improve water quality to treat these hazardous contaminants by using efficient and cost-effective methods.

Various methodologies have been employed to purify water contaminated with organic dyes. These methods include osmosis, adsorption, oxidation, ozonation, reduction, hydrogenation, anodic oxidation, coagulation, photodegradation, ion exchange, membrane filtration, biological treatment, electrochemical methods, and catalytic reduction.^16−21^ Unfortunately, most of the previously mentioned methods are pH-dependent, time-consuming, and entail high functioning costs. Additionally, these methods generate large disposal solutions and require the use of chemicals. However, the catalytic reduction technique has received significant attention due to its ease of application, cost-effectiveness, cleanliness, and ability to rapidly purify contaminated water from organic dyes. In this method, the toxic organic dyes are converted into less toxic or nontoxic products through a green, safe, and economical reducing agent, usually sodium borohydride (NaBH_4_) in the presence of a reusable solid catalyst that can facilitate organic dye reduction. The key role of the catalyst is to transfer electrons from electron donors to electron acceptors, resulting in an intermediate redox potential that can act as an electron relay to aid electron transfer.^16,22,23^

Various nanomaterials have been used as catalysts due to their unique physiochemical properties, as this process is favorable thermodynamically but unfavorable kinetically in the absence of a catalyst.^23−28^ These catalysts include silver nanoparticles (Ag-NPs),^29−36^ silver-copper oxide nanocomposites,^37^ modified Ag-NPs,^38^ gold nanoparticles,^39−41^ iron oxide nanoparticles,^42^ activated carbon,^43^ graphene oxide,^44,45^ zinc oxide nanoparticles,^46^ zirconium oxide,^47^ Cu/sodium borosilicate nanocomposite,^48^ Pd/calcium lignosulfonate nanocomposite,^49^ lignin-derived (nano)materials,^50^ palladium nanoparticles,^51,52^ and Cu NPs@Fe_3_O_4_.^53^ Nanomaterials can be generated through two primary approaches: the “top-down” and “bottom-up” methods. The top-down method reduces large units into nanosized structures, while the bottom-up method builds nanosized structures from small units. On the other hand, the methodologies of nanomaterial synthesis can be classified into biological, physical, chemical, photochemical, and electrochemical techniques.^54−57^ Although most of these methods produce clean and well-defined nanomaterials, most are expensive, time-consuming, and not eco-friendly due to the need to use toxic chemicals. However, the biological method is considered environmentally friendly, cost-effective, and scalable.^58−60^

Silver nanoparticles are nanostructured materials with particle sizes ranging between 1 and 100 nm. They have been utilized in various applications, such as chromatography, drug resistance, and biological applications like antimicrobial, micro-DNA, and leukemia–DNA detections.^61−66^ Furthermore, they have special importance in chemistry as heterogeneous catalysts in organic synthesis or water purification from organic pollutants. Although they can be generated by several means, the biological method is considered to be the best due to its low cost and environmental friendliness. Thus, Ag-NPs were prepared through a biosynthesis approach by various research groups using extracts from plants such as *Nervalia zeylanica*,^13^*Terminalia arjuna*,^67^*Artemisia oliveriana*,^68^*Solanum indicumlinn*,^69^*Persicaria odorata*,^22^*Caralluma acutangula*,^70^ and *Citrus macroptera* fruit.^71^ The Ag-NPs produced from all of the listed plant extracts varied in size and properties owing to the nature of phytochemicals in plant extracts, which control the nucleation of Ag-NPs from the solution.

This study investigates, for the first time, the green synthesis of Ag-NPs and their applications as antioxidants, antimicrobials, and catalysts for contaminated water remediation. The study aims to prepare Ag-NPs from *Acacia ehrenbergiana* plant cortex (AEPC) extract. The generation of Ag-NPs is accomplished in three main phases: activation, growth, and termination, through mixing an aqueous solution of silver nitrate with AEPC extract at 80 °C for 2 h. The shape and morphologies of the prepared Ag-NPs were studied through various analytical techniques, including UV–vis spectroscopy, Fourier transform infrared (FTIR) spectroscopy, thermogravimetric analysis (TGA), X-ray diffraction (XRD), scanning electron microscopy (SEM), transmission electron microscopy (TEM), and X-ray photoelectron spectroscopy (XPS). The as-prepared Ag-NPs were employed as a heterogeneous catalyst to reduce RhB dye from aqueous solutions in the presence of NaBH_4_ as a reducing agent. Therefore, to optimize catalytic reduction, various factors were investigated, such as the effect of Ag-NP concentration, the amount of NaBH_4_, the common-ion effect on RhB dye reduction, recyclability, and contact time. Furthermore, the prepared Ag-NPs were used as antibacterial and antifungal agents against 16 Gram-positive and Gram-negative bacteria and 1 fungal species. Owing to the ease of the synthesis strategy of Ag-NPs, they are a cost-effective starting material with high catalytic reduction efficiency and excellent antibacterial and antifungal activities.

## 2 Experiment Section

### 2.1 Materials and Chemicals

All chemicals, including silver nitrate (99.9%), methanol (99.5%), RhB (99.9%), sodium borohydride (99.9%), sodium chloride (NaCl), potassium chloride (KCl), and calcium chloride (CaCl_2_), were purchased from Sigma-Aldrich and used without further purification. The AEPC was obtained from southwest Saudi Arabia (Jazan province). Deionized (DI) water was used as a solvent in the Ag-NPs synthesis and RhB stock solution preparations. Laboratory-maintained cultures of *Staphylococcus aureus* (S1) and 12 strains of methicillin-resistant *S. aureus* (MRSA; S2–S13) were the 13 Gram-positive bacterial strains. *Escherichia coli* (S14), *Klebsiella pneumonia* (S15), and *Pseudomonas aeruginosa* (S16) were the Gram-negative bacteria used to study the antibacterial activity of the Ag-NPs. *C. albicans* (S17) was the fungus used to study the antifungal activity of the Ag-NPs.

### 2.2 Synthesis of the AEPC Extract

The AEPC was washed several times with DI water, then dried, and ground into a fine powder. Approximately 25 g of AEPC powder was dispersed in 600 mL of DI water. The mixture was then heated at 90 °C for 4 h until the solution’s color changed from colorless to orange. Finally, the solution was cooled down to room temperature, filtered, and stored at 4 °C in the fridge for further use.

### 2.3 Synthesis of the Ag-NPs

The green synthesis of the Ag-NPs was conducted as follows. First, 200 mL of the AEPC extract was heated to 80 °C and stirred for 30 min. Further, 10 mL of AgNO_3_ (2 mM) was slowly added to the above-mentioned extract solution in the dark. The reaction was carried out for 2 h to complete the nucleation and stabilization of the Ag-NPs. Finally, the black precipitate of the Ag-NPs was centrifuged and washed with DI water and methanol. The collected sample of Ag-NPs was further dried in the oven at 60 °C overnight.

### 2.4 Instrumentations

The as-synthesized Ag-NPs and AEPC extract were characterized using several analytical instruments. The UV–vis absorbance spectra were recorded using an SCO TECH SPUV-26 spectrophotometer (Germany) equipped with 1.0 cm quartz cuvettes, a D_2_ lamp as the UV source, and a W lamp as the visible light source. UV–vis diffuse reflectance spectra (DRS) were recorded using a Shimadzu UV-3600-DRS spectrophotometer (Japan). Fourier transform infrared (FTIR) spectra were recorded through the Shimadzu Prestige-21 IR spectrometer (Japan) and measured from 450 to 4000 cm^–1^. ζ-Potentials were measured using Malvern Instruments Nano ZS90, ZEN3690 (U.K.). Powder X-ray diffraction (XRD) patterns were measured at room temperature using the Shimadzu XRD, LabX-6000 XRD X-ray diffractometer (Kyoto, Japan) with a Cu Kα (λ = 1.54056 Å), working on 40/30 kV/Milli Ampere, at 2°/m amid 20–80° angles. X-ray photoelectron spectroscopy (XPS) analysis was done using a surface science instrument X-probe, X-Ray000 400 μm–FG ON (400 μm). Thermogravimetric analysis (TGA) was recorded using the Shimadzu DTG-60H simultaneous DTA-TG Apparatus (Kyoto, Japan). The analysis was completed in a nitrogen environment at a heating rate of 5.0 °C min^–1^. Transmission electron microscopy (TEM) images were obtained at 120 kV with the JEOL HRTEM, JEM-2100F-Tokyo (Japan). Scanning electron microscopy (SEM) images were taken using the Hitachi SU-70 field emission scanning electron microscope with an energy of 5.0 kV. Energy-dispersive X-ray spectrometry (EDX) and atomic mapping were done with the Quanta FEG 250 SEM with a field emission gun (the Netherlands). Gas chromatography–mass spectrometry (GC-MS) analysis was conducted using a GC-MS machine (QP2010 Ultra, Shimadzu Corporation, Kyoto, Japan). The separation was achieved on an Rtx-5MS capillary column (30 m length × 0.25 mm diameter) coated with a 0.25 μm film thickness stationary phase (Restek Corporation, U.S). Helium was employed as the carrier gas at a constant linear velocity of 36.3 cm/s. A sample volume of 1.0 μL was injected using an AOC-20i+s autoinjector. The gas chromatography (GC) oven temperature program started at 50 °C for 1 min, heated at 5 °C/min to 300 °C, and held for 10 min. The temperatures of the GC injection port, MS ion source, and interface were set at 290, 230, and 280 °C, respectively. Full scan mass spectra were recorded within a range from *m*/*z* 50 to 500. The GC-MS solution software and the NIST 11 mass spectral library were employed for data processing and compound identification, respectively.

### 2.5 Catalytic Reduction Experiments

The catalytic reduction of RhB dye in the presence of NaBH_4_ using Ag-NPs was performed in a quartz UV cuvette. Briefly, 100 μL of 1.0 mM RhB solution was added to 2.0 mL of DI water and 1.0 mL of 10.0 mM NaBH_4_ solution. Then, 20 μL of 0.05% Ag-NPs aqueous suspension was added to the reaction mixture. Different concentrations of the RhB dye were analyzed at different times and measured by UV–vis spectroscopy. The strong visible region absorption of RhB (λ_max_, at 554 nm) dye and the linear correlation with absorbance and reduction were measured according to the Beer–Lambert law. The catalytic reduction percentage (CR%) was calculated using eq 1, which involved measuring the concentration of RhB dye before (*C*_0_) and after adding the Ag-NPs (*C_t_*) as time progressed.^72,73^
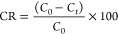
1To optimize the catalytic reduction of RhB dye, the effect of pH was studied by adjusting the pH of 16 ppm RhB dye solutions between 2 and 10 using 0.1 M HCl or 0.1 M NaOH. The effect of concentration was investigated through 8, 16, and 24 ppm RhB dye solutions at pH = 5 and 25 °C. The effect of the Ag-NPs dose was studied using 20, 30, 40, and 50 μL of the Ag-NPs. Similarly, the effect of the NaBH_4_ dose was studied by using 10, 25, 50, and 75 μL of the NaBH_4_. To elucidate the applicability of Ag-NPs in the complex mixture of RhB dye, the effect of common ions was examined in the presence of Na^+^, K^+^, and Ca^2+^ ions for 16 ppm RhB solutions. The reduction was determined using a UV–vis spectrophotometer.

### 2.6 Ag-NPs Reusability

Appropriate amounts of Ag-NPs and NaBH_4_ were applied to a 100 mL solution of RhB dye to reduce the dye. The reduction of RhB was done at room temperature in the dark until the RhB dye solution became colorless. The Ag-NPs were separated by centrifuge, washed with DI water and methanol, and dried in the oven at 60 °C. From the dried Ag-NPs, a 0.05% suspension was prepared and applied for cycle 1 in the UV cuvette. The remaining Ag-NPs were reused for a major reaction in the dark. Similarly, cycle 4 was checked for the reusability of Ag-NPs, as shown in Scheme S1 in the Supporting Information.

### 2.7 Antimicrobial Activity Analysis

The bacteria were grown on Mueller and Hinton agar at 37 °C. Molecular identification by 16 DNA gene sequencing was done for some selected strains, mainly *S. aureus* (S1), *E. coli* (S14), and *K. pneumonia* (S15); all other samples were identified using standard biochemical tests.^74^ The antibacterial and antifungal activities of the synthesized Ag-NPs were studied against bacterial and fungal isolates by using the agar well diffusion assay with Muller and Hinton agar (MHA). A 24 h fresh culture of the test isolates (S1–S17) was used to prepare a saline suspension to match the 0.5 McFarland turbidity standard tubes (1.5 × 10^8^ colony forming units/mL). Each isolate was spread on sterile MHA plates using sterile cotton swabs. Wells were dug in each plate using a sterile metal cork borer. Six different concentrations of the Ag-NPs were used: 30, 25, 20, 15, 10, and 5 mg. Then, 100 μL of each concentration was inoculated in each corresponding well. Standard antibiotic disks of Vancomycin (30 μg), Gatifloxacin (5 μg), and Rifamycin (5 mg) were used as the control for comparison. The inoculated plates were kept in an incubator at 37 °C for 24 h. The zone of inhibition was measured in millimeters (mm) using a zone-measuring ruler (Himedia, India). Minimum inhibition concentration (MIC) was calculated as the lowest concentration showing a zone of clearance. All of the test procedures confirmed the recommended standards of the Clinical and Laboratory Standards Institute (CLSI, 2000), and all of the tests were done in triplicate.

## 3 Results and Discussion

### 3.1 Ag-NPs Design Strategy

**Scheme 1:**
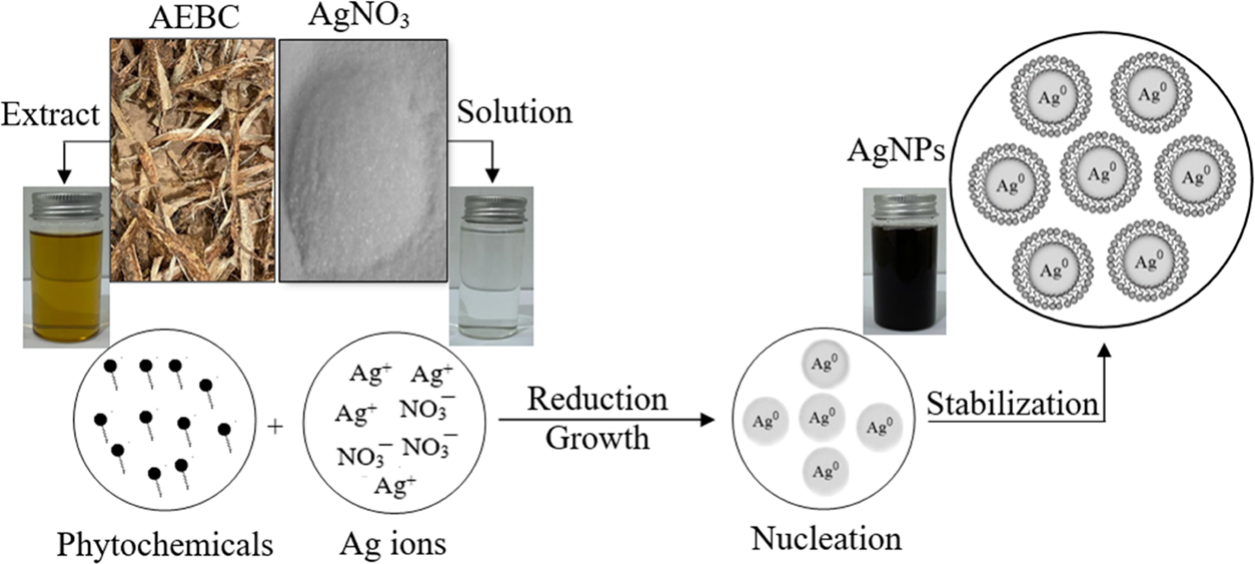
General Synthetic Strategy to Prepare Ag-NPs from AEPC Extract

The rapid generation of Ag-NPs via an eco-friendly method using AEPC extract was accomplished by mixing an aqueous solution of silver nitrate with AEPC extract at 80 °C. The formation of Ag-NPs was indicated by the color change of the AEPC extract from yellow to black within 2 h when the two solutions were mixed. The changes in solution colors were attributed to the reduction of silver ions in the solution into silver nanoparticles through the existing phytochemicals in the AEPC extract.^13,29,39^ The proposed mechanism of Ag-NPs formation from AEPC extract is depicted in Scheme 1 in three main steps. The first step, known as the activation, involves the reduction of Ag^+^ to Ag^0^, which leads to the nucleation of reduced silver atoms. The second step is the growth of uniform structure and further reduction of silver ions within the increasing thermodynamic stability of the formed Ag-NPs. The last step is the stabilization of the Ag-NPs through the phytochemicals in the AEPC extract, which work as capping agents and prevent the agglomeration of the Ag-NPs.^75,76^ Compared to other methods, the preparation of Ag-NPs from AEPC extract is novel due to the phytochemicals in the extract that can act simultaneously as reducing and capping agents. In addition to this, the method is an eco-friendly, easy, fast, and mild-condition synthetic strategy.

### 3.2 AEPC Extract and the Ag-NPs Characterizations

**Figure 1:**
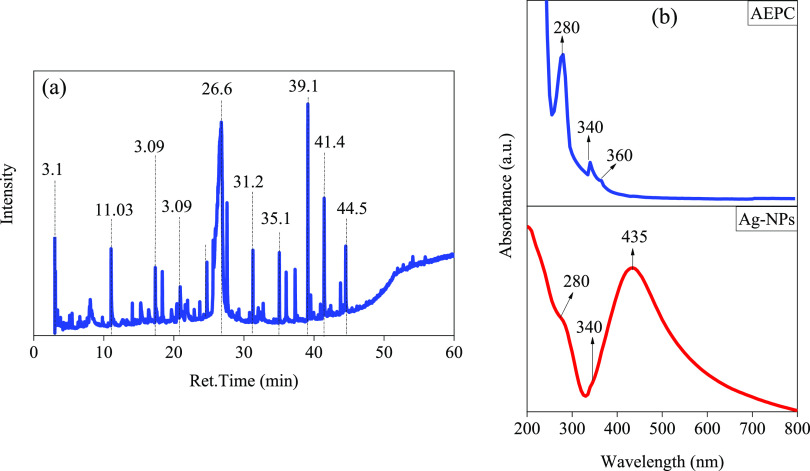
(a) GC-MS chromatogram of the AEPC extract and the corresponding components are shown in Table S1. (b) UV–vis spectra of the AEPC extract and Ag-NPs.

To identify the phytochemicals in the AEPC extract that facilitate both reduction and capping of Ag-NPs formation in the AEPC extract, GC-MS analysis was employed. The results are plotted in Figure 1a and show approximately 25 different phytochemical compounds present in the AEPC extract. Table S1 summarizes the IUPAC names of the phytochemicals and provides a quantitative analysis of their relative amounts in the AEPC extract. All major phytochemicals in the AEPC extract commonly have either an aromatic π-system or are functionalized by heavy donor atoms such as oxygen, nitrogen, sulfur, and chlorine in different functional groups. Therefore, the phytochemicals in the AEPC extract can donate their electrons to silver ions and cause the reduction of Ag^+^ to Ag^0^, generating stabilized Ag-NPs, which is also in good agreement with reported articles in the literature.^13,39,70,77^ The formation of Ag-NPs was further examined using UV–vis spectroscopy, and the spectra are plotted in Figure 1b for both the AEPC extract and Ag-NPs. The results showed that both materials have two absorption peaks at 280, 340, and 360 nm, which are attributed to the (π to π*) and (n to π*) electronic transitions in the phytochemicals in the AEPC extract.^73,78^ These absorption bands are characteristic of phytochemicals, such as phenols, alkaloids, saponins, flavonoids, and terpenoids, as mentioned in previous studies.^79^ However, the Ag-NPs UV–vis spectrum shows one more peak at 435 nm that can be attributed to the surface plasmon resonance (SPR) of the Ag-NPs.^13,76^ UV––vis absorption peaks in both the AEPC extract and Ag-NPs indicate the role of phytochemicals as a capping agent to prevent the aggregation of Ag-NPs. Furthermore, the stability of the Ag-NPs from the AEPC extract was investigated by measuring the intensity of the SPR peak over time, and the spectrum is plotted in Figure S1. The results revealed that 120 min was sufficient to generate stable Ag-NPs due to the complete formation of nanoparticles in the solution.

**Figure 2:**
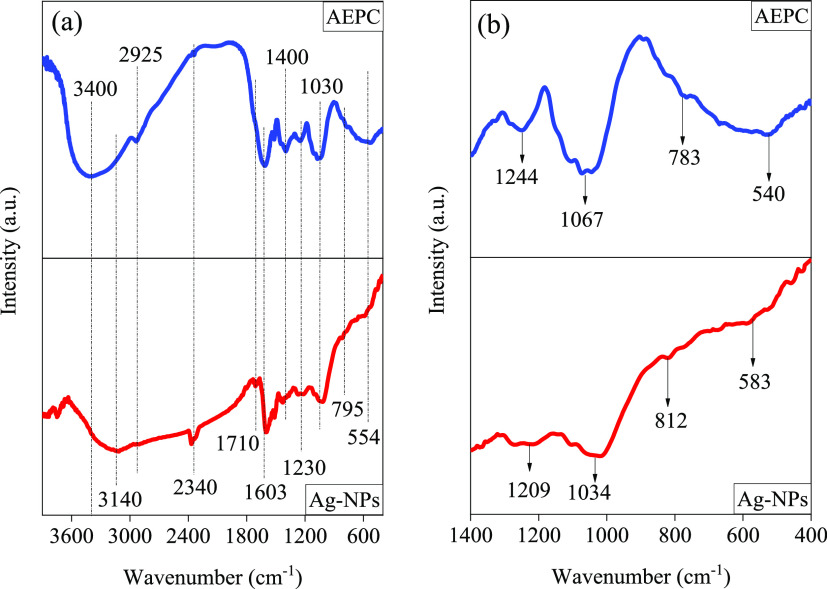
FTIR spectra of the AEPC extract and Ag-NPs. (a) Wavenumbers between 400 and 4000 cm^–1^. (b) Wavenumbers between 400 and 1400 cm^–1^.

To identify the functional groups present in both the AEPC extract and Ag-NPs, FTIR analysis was employed for both samples, and the results are depicted in Figure 2a. It is clear to see that both samples have common peaks at 554, 795, 1030, 1230, 1400, 1593, 1700, 2340, and 3140–3400 cm^–1^, which can be attributed to the aromatic rings, C–H bending, C–O esters, C–O carboxylic acid, C–H bending, C=C stretching, C=O stretching, H–O carboxylic acid stretching, aliphatic C–H stretching, and H–O carboxylic acid stretching, respectively.^39,71^ To obtain specific information about Ag-NPs stabilization by phytochemicals in the AEPC extract, Figure 2b compares the region from 500 to 4000 cm^–1^ of the FTIR spectra of both the AEPC aqueous extract and Ag-NPs. It is clear to see that the AEPC extract exhibits two peaks at 540 and 783 cm^–1^ due to alkyl halide (C–Cl) stretching that shifted to 583 and 812 cm^–1^, respectively, for stabilized Ag-NPs. Similarly, in the AEPC extract spectrum, the C–O bond stretching from carbohydrate and carboxylic acid was observed at 1067 and 1244 cm^–1^, respectively. In the stabilized Ag-NPs spectrum, these peaks shifted to 1034 and 1209 cm^–1^, respectively.^39,71,80^ Furthermore, the broadband in the AEPC extract at 3400 cm^–1^ was attributed to the (−OH) carbohydrate proteins and polyphenols, which shifted to a lower wavelength at 3140 cm^–1^ in capped Ag-NPs. These shifts confirmed the role of polyphenolic compounds in reducing the Ag^+^ ions into Ag-NPs in addition to the role of phytochemicals in stabilizing the nanoparticles.^81,82^ Therefore, it can be concluded from the FTIR analyses that the presence of the functional groups in both the AEPC extract and Ag-NPs can be used as good evidence to prove the role of phytochemicals as a capping agent to prevent the aggregation of Ag-NPs.

**Figure 3:**
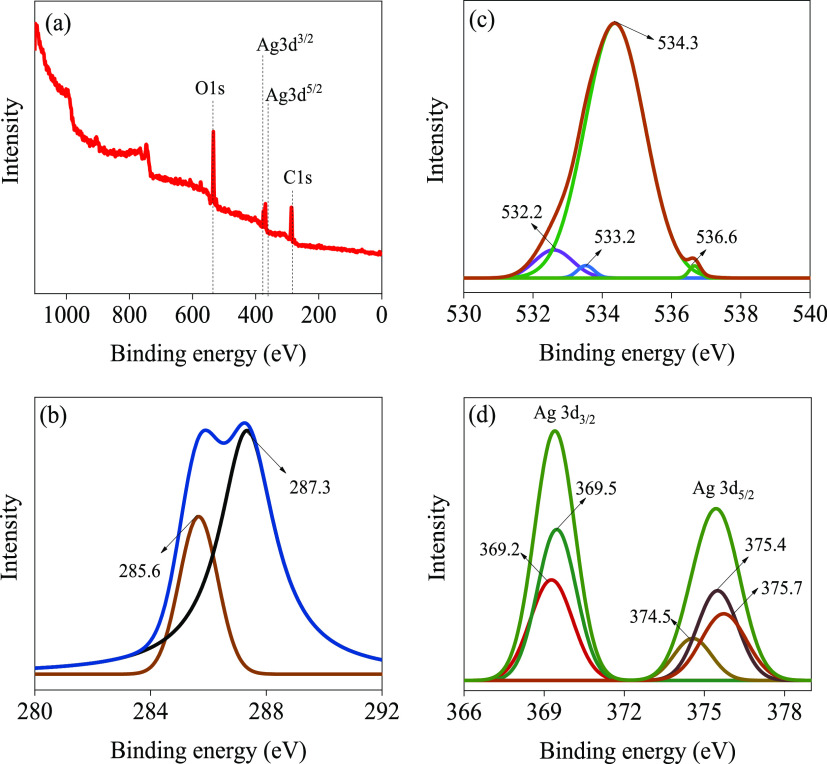
XPS spectra of green-synthesized Ag-NPs: (a) survey spectrum, (b) C 1s, (c) O 1s, and (d) Ag 3d high-resolution spectra.

To confirm the FTIR results, XPS analysis was employed, and the results are plotted in Figure 3. The survey spectrum in Figure 3a shows four peaks due to C 1s, Ag 3d, and O 1s. The C 1s high-resolution spectrum in Figure 3b deconvolutes into two peaks at the electron binding energies of 287.3 and 285.6 eV due to C=O and C–O, respectively.^45^ The O 1s high-resolution spectrum in Figure 3c displays four peaks at the electron binding energy values of 536.7, 533.4, 536.7, and 537.7 eV due to Ag–O, C=O, C–O, and C–O, respectively. The Ag 3d in Figure 3d has two peaks at the electron binding energy values of 369.5 and 374.5 eV, corresponding to the spin-orbit splitting of Ag 3d_5/2_ and Ag 3d_3/2_, respectively.^83^ Furthermore, there are three peaks at 370.5, 372.5, and 376.8 eV that can be attributed to the presence of oxidized species of Ag, which is also in good agreement with the literature.^83,84^ Therefore, it is clear that the XPS analysis confirms the FTIR data by the presence of the organic phytochemicals that work as stabilizers.

**Figure 4:**
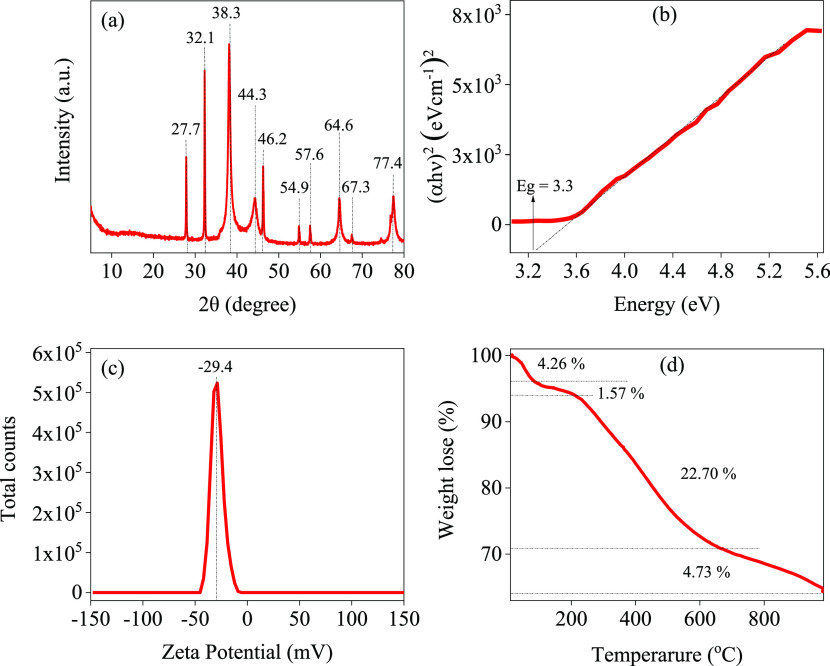
(a) XRD, (b) estimated band gaps using the Touk equation, (c) ζ-potential, and (d) TGA of the Ag-NPs.

The XRD spectroscopy was used to investigate the crystallinity of the prepared Ag-NPs, and the results are presented in Figure 4a. The results indicate clear crystallinity of the Ag-NPs evidenced by the presence of sharp XRD peaks at 2θ values of 27.7, 23.1, 38.3, 44.3, 46.2, 54.9, 57.6, 64.6, 67.3, and 77.4° in the spectrum. The peaks at 38.3, 44.3, 64.6, and 77.4° were assigned to the (111), (200), (220), and (311) diffraction planes of the Ag-NPs, respectively. However, the appearance of the other peaks could be attributed to the crystal diffraction planes of silver oxide, which might be covered on the surface of the Ag-NPs, or to organic phytochemicals in the leaf extract that act as capping agents. Therefore, the presence of the (111) diffraction planes with the highest peak intensity demonstrates the formation of the Ag-NPs.^13,85−87^ Nevertheless, the presence of the other diffraction planes provides additional evidence to support the role of the phytochemicals as capping agents, which is consistent with the FTIR and UV–vis spectral analyses.^67,79^

The optical direct band gap of the prepared Ag-NPs was calculated using the Tauc plot, as depicted in Figure 4b. The results showed that Ag-NPs have a higher band gap (3.3 eV) than the most reported values in the literature, which may be attributed to quantum confinement. The potential stability of the Ag-NPs was examined using ζ-potential, and the result of −29.4 mV is plotted in Figure 4c. The synthesized Ag-NPs from the AEPC extract showed a negative charge and were stable at room temperature. The thermal stability of the Ag-NPs was studied using TGA, the results of which are plotted in Figure 4d. The graph shows four thermal decomposition processes at 100, 250, 620, and 900 °C, with weight loss of 4.26%, 1.57%, 22.70, and 4.73%, respectively. These can be attributed to the loss of water, volatile molecules, organic biomolecules, and resistant aromatic compounds on the surface of the Ag-NPs, respectively. Therefore, the TGA results demonstrate the role of the phytochemicals as capping agents and are also in good agreement with the FTIR, UV–vis, and XRD analyses as well as the literature.^31,88,89^

**Figure 5:**
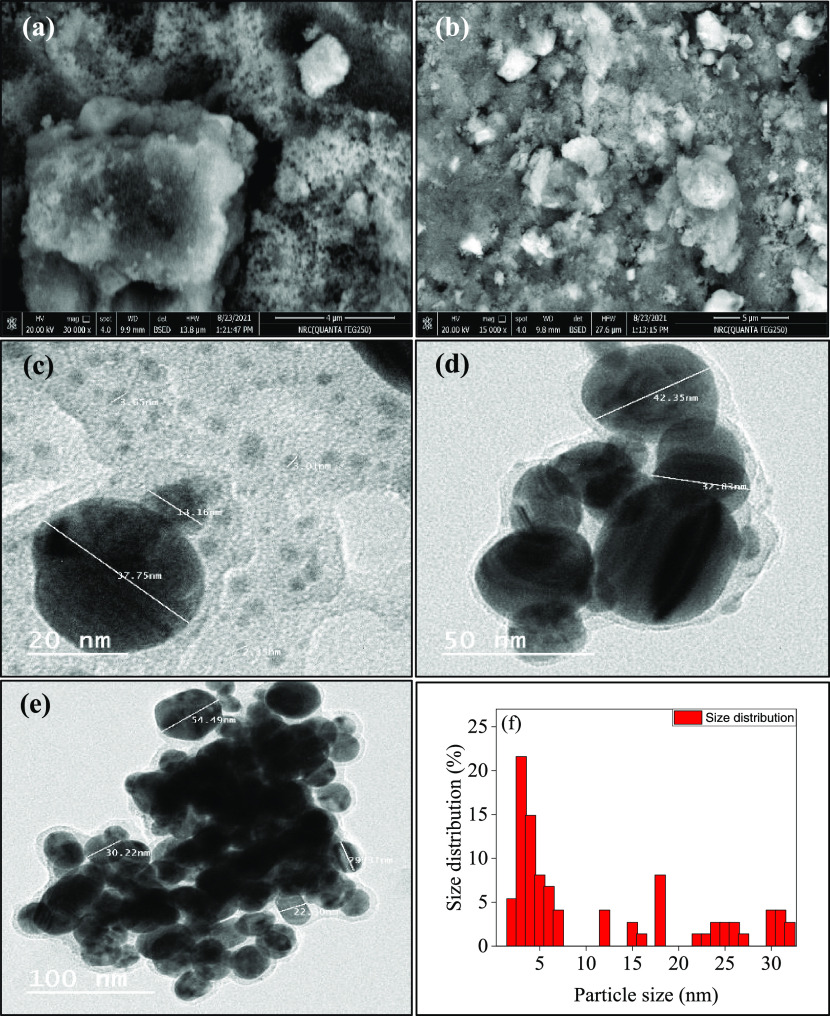
(a, b) SEM images, (c–e) TEM images, and (f) particle size distribution of the Ag-NPs.

The morphologies and sizes of the prepared Ag-NPs were investigated using SEM and TEM microscopes. The SEM images in Figure 5a,b show that the Ag-NPs are homogeneously spherical, and the surface of the particles is covered with an organic layer from the phytochemicals, which act as capping agents. Moreover, the TEM images in Figure 5c–e reveal the spherical morphology of the Ag-NPs with small groups of particles that were also observed due to accumulation during sample preparation. Additionally, the size of the Ag-NPs was calculated by TEM and the Debye–Scherrer eq S1. The results showed that the particle sizes ranged from 1 to 40 nm, as shown in Figure 5f and Table S2, respectively. The elemental composition of the Ag-NPs is displayed in Figure S2 and Table S3 using EDX analysis. The results show that the sample contains 59.11% silver and 19.74, 16.07, 4.3, and 0.77% of C, O, Cl, and S, respectively. The results suggest that phytochemicals play a role as capping agents, as evidenced by the lower amounts of other elements present and the highest weight percentage of Ag in the sample. Therefore, it can be concluded from all of the characterization analyses that the Ag-NPs capped with phytochemicals in the AEPC extract have a particle size distribution between 1 and 40 nm.

### 3.3 Catalytic Reduction of RhB

**Figure 6:**
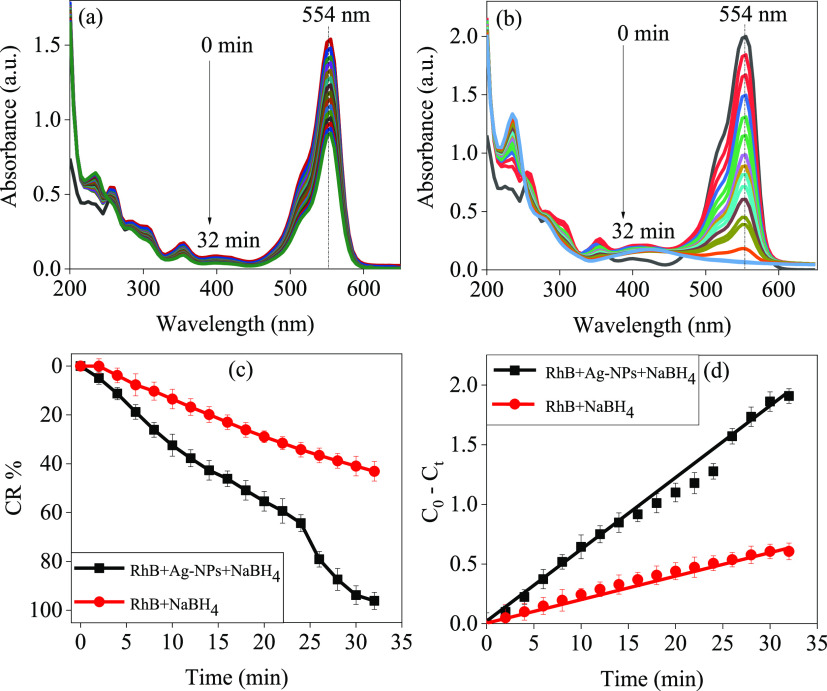
UV–vis absorption spectra of RhB dye during 32 min catalytic reduction using (a) without Ag-NPs, (b) with Ag-NPs, (c) calculated CR % of RhB, and (d) zero-order kinetic model of RhB CR [conditions: pH = 5, *C*_0_ = 16 ppm, *t* = 32 min, *T* = 298 K].

The as-prepared Ag-NPs were used as a catalyst to remediate contaminated water from RhB organic dye due to its detrimental nature to the environment. The UV–vis absorption spectra of the RhB as a function of time in the absence and presence of the Ag-NPs are depicted in Figure 6a,b, respectively. The results revealed that the catalytic reduction of the RhB in the presence of the Ag-NPs was completed within 32 min, while the catalytic reduction in the absence of the catalyst was very slow. To gain more details, the CR% was quantified using eq 1, and the results are plotted in Figure 6c. The results revealed that approximately 96 and 43% of the RhB dye was reduced within 32 min with and without the Ag-NPs, respectively. Numerous articles in the literature report that the unique low volume-to-high surface area ratio of Ag-NPs makes them effective catalysts, resulting in an increased rate of RhB dye reduction and degradation.^72,90^

To explain the experimental data of the catalytic reduction, the zero-order kinetic module was used, as the linear form of this module is represented by the following equation^70,72^
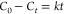
where *k* is the rate constant (mol L^–1^ min^–1^) that can be calculated from the slope of the line, *t* is the reaction time (min), and *C*_0_ and *C*_*t*_ are the initial and final concentrations (mol L^–1^) of the RhB dye before and after catalytic reduction, respectively. The results shown in Figure 6d fit quite well with the experimental data (correlation coefficient *R*^2^ > 0.98) to follow the zero-order chemical kinetic module. Table 1 displays the calculated rate constant for the catalytic reduction of RhB using Ag-NPs in the presence of NaBH_4_ as a reducing agent, which is 0.059 mol L^–1^ min^–1^. This value is higher than the rate constant observed in the absence of Ag-NPs (0.019 mol L^–1^ min^–1^). Furthermore, Table S4 compares the rate constant of this study to the rate constants of organic dye catalytic reduction using NaBH_4_ as a reducing agent with Ag-NPs prepared from *C. acutangular* plant extract in the literature.^70^ It is clear that the Ag-NPs catalyst in this study’s synthetic strategy is faster than other catalysts. The high catalytic reduction efficiency could be attributed to the unique synthetic strategy of the Ag-NPs from the AEPC extract, as the phytochemicals can facilitate both the reduction and capping of Ag-NPs. Therefore, very fine Ag-NPs sizes with high surface areas act as an efficient catalyst to eject electrons from NaBH_4_ into the surface of the Ag-NPs, which will be gained by the RhB dye and cause reduction.

**Table 1:** Kinetic Constants of RhB Dye Photodegradation Reduction by Ag-NPs

catalyst	regression equation	*k* (mol L^–1^ min^–1^)	*R*^2^
RhB + NaBH_4_	*y* = 0.0194*x* – 0.0348	0.019	0.9885
RhB + Ag-NPs + NaBH_4_	*y* = 0.059*x* – 0.0028	0.059	0.9881

### 3.4 RhB Catalytic Reduction Optimizations

**Figure 7:**
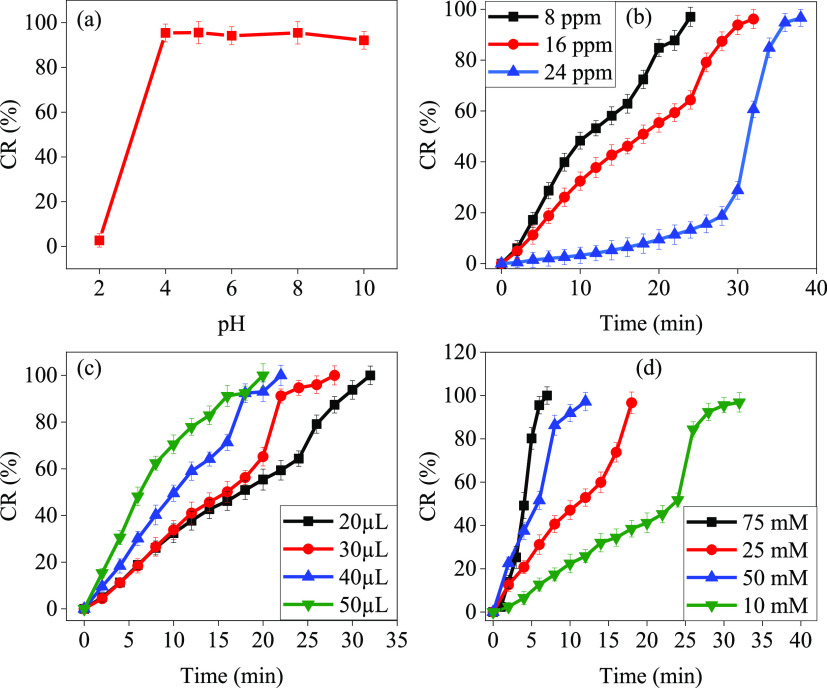
(a) Effect of the pH [conditions: *C*_0_ = 16 ppm, dose = 20 μL of 0.05% Ag-NPs, and 1 mL of 10 mM NaBH_4_]. (b) Effect of the RhB concentration [conditions: pH = 5, dose = 20 μL of 0.05% Ag-NPs, and 1 mL of 10 mM NaBH_4_]. (c) Effect of the Ag-NPs dosage [conditions: pH = 5, *C*_0_ = 16 ppm, and 1 mL of 10 mM NaBH_4_]. (d) Effect of the NaBH_4_ dosage [conditions: pH = 5, *C*_0_ = 16 ppm, and dose = 20 μL of 0.05% Ag-NPs], on the RhB.

To optimize the conditions for RhB dye catalytic reduction, the effects of pH, RhB dye concentration, NaBH_4_ concentration, and Ag-NP concentrations were investigated. The effect of pH was studied at different pH levels (2, 4, 5, 6, 8, and 10) while keeping the concentration, time, dosage, and temperature constant. This is because pH plays a critical role in facilitating RhB adsorption onto the surface of Ag-NPs, thus promoting dye catalytic reduction. The results depicted in Figure 7a indicate that RhB dye catalytic reduction significantly increased when the pH of the solutions increased from 2 to 10, reaching maximum values at pH > 4. As reported in the literature, these results can be explained by the negative ZPC of the Ag-NPs, where the positively charged RhB dye in an acidic solution may have a higher tendency to aggregate on the negatively charged Ag-NPs surface.^91^ The effect of RhB dye concentrations on CR was also investigated by using 8, 16, and 24 ppm RhB solutions while keeping pH, time, dosage, and temperature constant. The results in Figure 7b reveal that as the dye concentration increased, the required time to reduce the dye also increased to be 24, 32, and 38 min for 8, 16, and 24 ppm RhB solutions, respectively. The catalytic reduction percentages of 1 mM in 2 mL of DI water of RhB and 1 mL of 10 mM NaBH_4_ solution were studied using different dosages of 0.05% Ag-NPs (20, 30, 40, and 50 μL) and are plotted in Figure 7c. The results indicate that as the amount of Ag-NPs increased, the time required to reduce RhB dye decreased to 32, 26, 20, and 18 min, respectively. This could be attributed to the presence of more active catalytic sites when the concentration of Ag-NPs increases, therefore, facilitating the reduction of RhB dye. Similarly, the catalytic reduction percentage results of 1 mM in 2 mL of DI water of RhB and 20 μL of 0.05% Ag-NPs with different concentrations of NaBH_4_ (10, 25, 50, and 75 mM) are depicted in Figure 7d. The results revealed that the time required to achieve 96% CR decreased to 32, 18, 12, and 7 min when the amount of NaBH_4_ was 10, 25, 50, and 75 mM, respectively. As reported in various literature, this could be attributed to the presence of more active catalytic sites when the concentration of Ag-NPs increases, as well as the high surface area, which can increase the reduction rate of RhB dye.^70,90^

### 3.5 Catalyst Stability and Reusability Test

**Figure 8:**
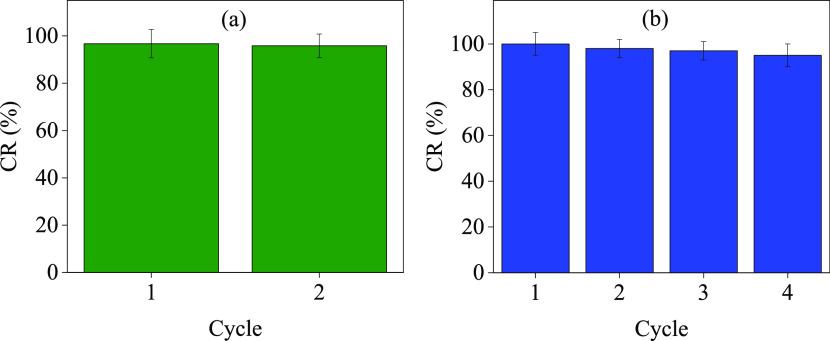
(a) Stability test and (b) reusability test of the Ag-NPs.

The effect of the presence of other interfering ions was studied in the presence of Na^+^, K^+^, and Ca^2+^ as common ions in water, and the results are plotted in Figure 8a. The results showed that the catalytic reduction of the RhB dye in cycle 1 resembles the results in the presence of interfering ions in cycle 2. Therefore, it can be concluded that the presence of Na^+^, K^+^, and Ca^2+^ as common ions does not affect RhB dye catalytic reduction by using Ag-NPs as a catalyst. The reusability cycles were performed to assist the stability and durability of the synergistic effect in degrading the RhB dye in the presence of NaBH_4_. Figure 8b shows that the Ag-NPs are stable and revealed up to four cycles for RhB dye reduction with a high synergistic effect in the presence of NaBH_4_. However, the Ag-NPs efficiency effect decreases a small percentage for RhB dye reduction. However, efficacy is high due to the synergistic effect. The stability of the catalyst was examined by SEM and TEM images in Figures S4a and S4b, respectively. The images showed stable nanoparticles after three catalytic reduction cycles, which indicates the high stability of Ag-NPs.

### 3.6 RhB Catalytic Reduction Mechanism

**Scheme 2:**
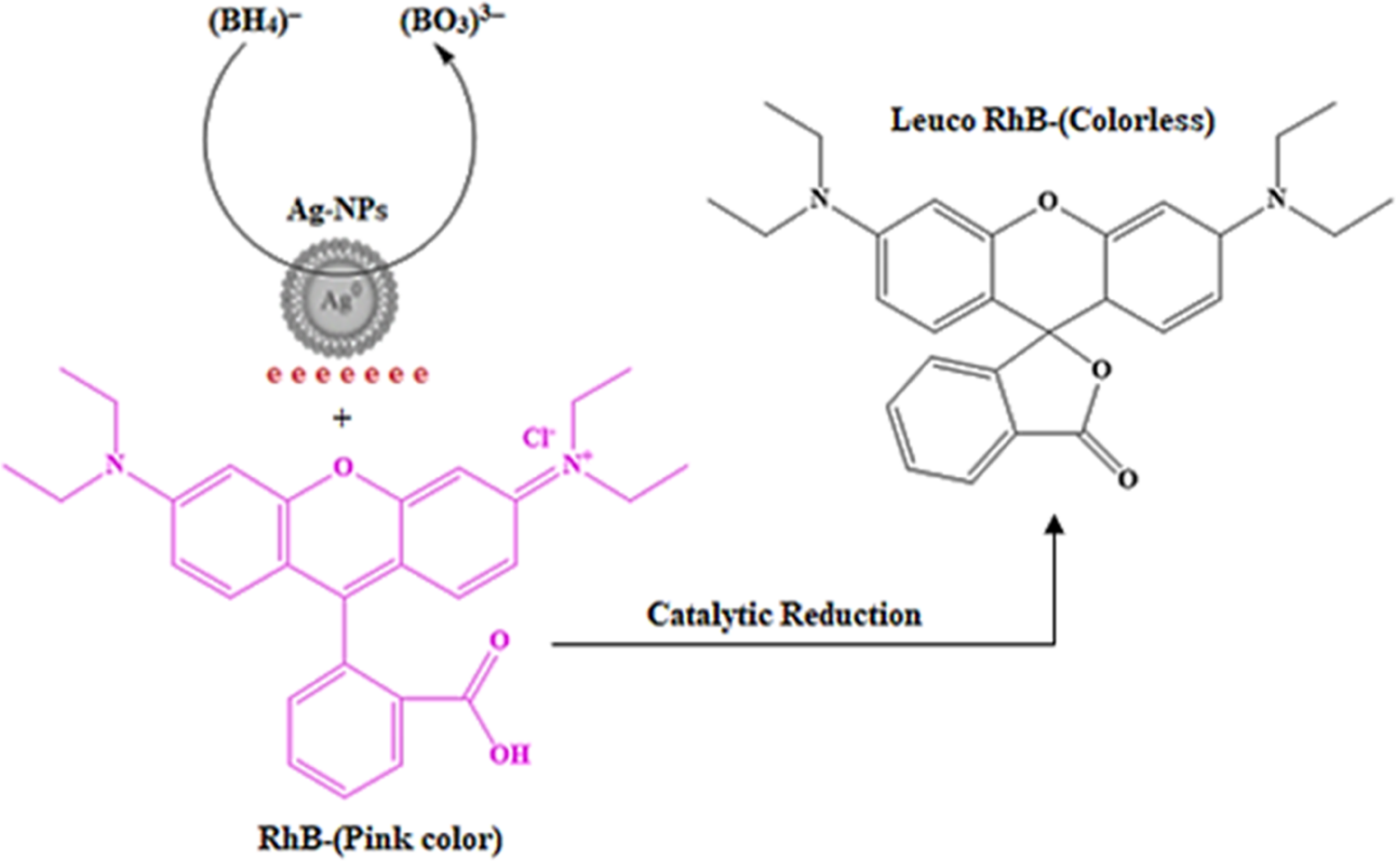
Proposed Mechanism for RhB Dye Catalytic Reduction by Ag-NPs Catalyst in the Presence of NaBH_4_ as a Reducing Agent

The rapid reduction of the RhB dye in the Ag-NPs and NaBH_4_ system relies on electrons. Scheme 2 shows the proposed reaction mechanism. In this mechanism, sodium borohydride acts as an electron donor, while RhB dye acts as an electron acceptor. However, electron transfer between them is hindered by the high differences in their redox potentials. Nonetheless, these processes become thermodynamically and kinetically favorable in the presence of Ag-NPs due to their intermediate reduction potential between sodium borohydride and RhB dye. The catalytic reduction reaction begins by adsorbing both the electron donor and acceptor on the surface of the Ag-NPs through electrostatic attraction with the phytochemical on the Ag-NPs surface. After that, the electrons are ejected from NaBH_4_ to the surface of the Ag-NPs and then acquired by the RhB dye, which causes the reduction to change into a colorless Leuco form.^68,72^

To demonstrate the strong potential of using the Ag-NPs to remediate contaminated water with organic dyes, Table 2 compares our green-synthesized Ag-NPs with past reported works. Green synthesis of Ag-NPs based on AEPC was observed to efficiently reduce the RhB dye within 32 min, compared to reported works in previous studies. The AEPC-based Ag-NPs surface functionality and stabilization assimilate the applications that are enhanced due to phytochemicals from the aqueous extract of AEPC. However, most of the preparation methods for Ag-NPs in the literature are time-consuming, expensive, and result in nanoparticle instability during long cycle runs. On the other hand, the AEPC-based Ag-NPs are economical, less time-consuming to synthesize, and serve as effective catalysts for RhB dye reduction and antibacterial applications in contaminated water.

**Table 2:** Various Organic Pollutants Remediation Using Ag-NPs Prepared through Different Reported Methods

S. No.	nanoparticles and synthesis	size (nm)	methodology for dye degradation	target dye (degradation)	time (min)	reference
1	Ag*-*ZnO/chemical	5	photocatalysis	rhodamine B (100%)	100	(3)
2	Ag/*Chlorella vulgaris*	55	photocatalysis	methylene blue (96.51%)	180	(92)
3	Ag/*Cyperus pangorei*	32–60	photocatalysis	rhodamine B (86%)	120	(57)
4	Ag/*Carica papaya*	10–70	photocatalysis	blue CP (90%)	30	(93)
5	Ag/*Ocimum tenuiflorum*	25–30	photocatalysis	sulforhodamine B (87%)	180	(94)
6	Ag/*C. acutangula*	1–30	synergistic effect with NaBH_4_	methylene blue (96.72%)	32	(79)
7	Ag/*T. arjuna*	10–50	synergistic effect with NaBH_4_	MB (93.60%)	20	(67)
				CR (92.20%)		
8	Ag/*Citrus paradisi*	10–50	synergistic effect with NaBH_4_	4-NP (88.90%)	9	(77)
				RhB (60.53%)	18	
9	Ag/AEPC	1–40	synergistic effect with NaBH_4_	rhodamine B (96%)	32	present work
10	AEPC extract		synergistic effect with NaBH_4_	rhodamine B (2%)	32	

### 3.7 Antimicrobial Analysis

**Figure 9:**
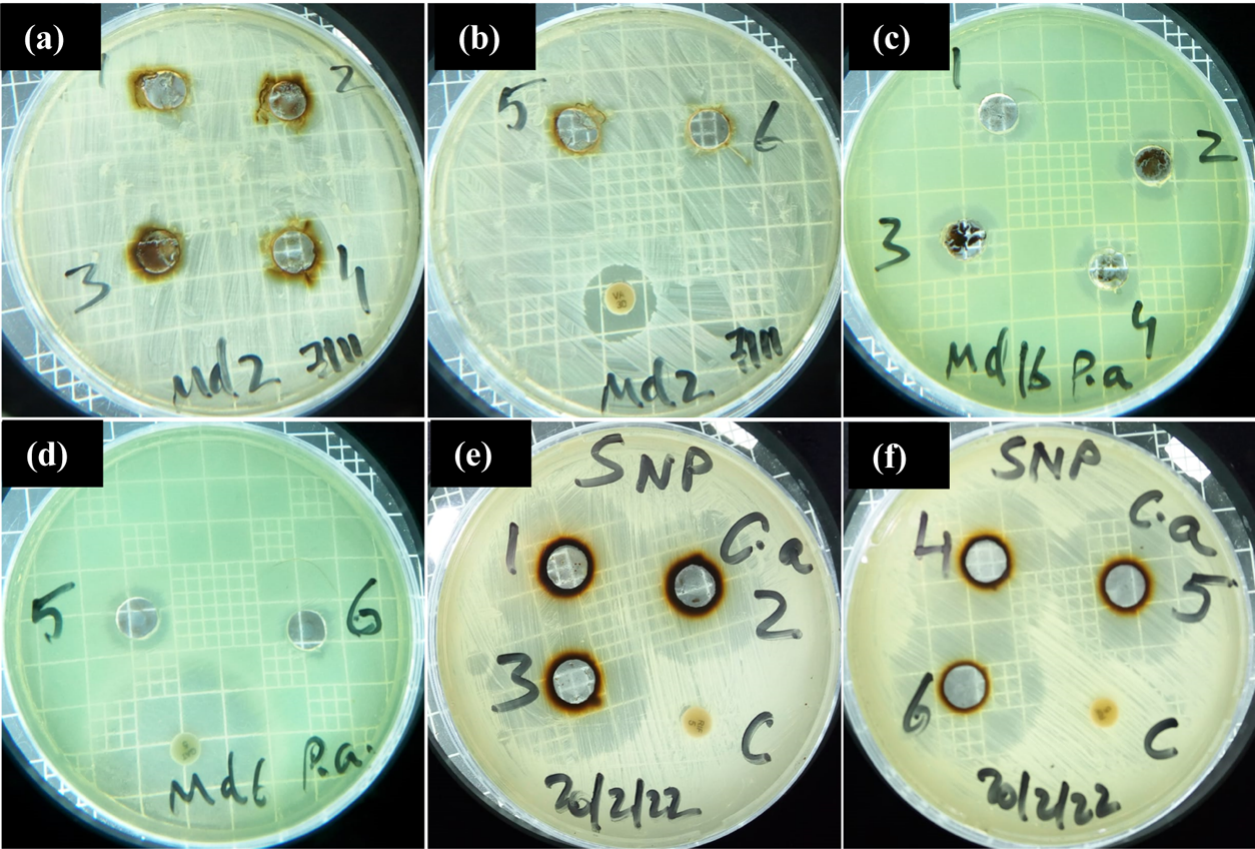
Antibacterial activity of the Ag-NPs against MRSA isolate S2 (a, b), *P. aeruginosa* isolate S16, (c, d) and *C. albicans* isolate S17 (e, f) on MHA. Concentrations in well 1 to well 6 (30, 25, 20, 15, 10, and 5 mg), respectively.

The prepared Ag-NPs were studied against different microbial isolates (16 bacterial and 1 fungal isolate), and the MIC results are presented in Figure 9 and Table 3. The results reveal that the S1, S2, S3, S5, S8, S9, S10, S12, S13, S15, and S16 strains have MIC values of 5 μg/mL. On the other hand, the MRSA strain (S7) and *E. coli* (S14) strains showed MIC values of 10 μg/mL, and there is no inhibition for two MRSA strains, such as S6 and S11 strains, even when applied with a high dosage of Ag-NPs (30 μg/mL). This might be because MRSA strains (S6 and S11) contain a highly defensive system and eventually grow even in the presence of Ag-NPs. The fungal isolate, *C. albicans* (S17), also exhibited an MIC value of 5 μg/mL. These results demonstrate the powerful antibacterial and antifungal activity of the prepared Ag-NPs, attributed to their broad-spectrum inhibition of several microorganisms. The high antimicrobial activities of the Ag-NPs could be attributed to their small particle sizes and high surface area, which can facilitate their attachment to microorganism cell wall surfaces. Several phytochemicals from AEPC are present on the Ag-NPs surface, directly interacting with the bacteria or fungal cell wall proteins, causing cell rupture and inhibiting growth. This finding is in good agreement with several reported articles in the literature, as shown in the comparison table (Table S5) with this study’s Ag-NPs.^95−99^

**Table 3:** Minimum Inhibitory Concentration of the Ag-NPs against Microbial Isolates

	microorganism		silver nanoparticle
No.	isolates	sample origin	MIC (μg/mL)
S1	*S. aureus*	human	5
S2	MRSA	human	5
S3	MRSA	human	5
S4	MRSA	human	25
S5	MRSA	human	5
S6	MRSA	human	no inhibition
S7	MRSA	human	10
S8	MRSA	human	5
S9	MRSA	human	5
S10	MRSA	human	5
S11	MRSA	human	no inhibition
S12	MRSA	human	5
S13	MRSA	human	5
S14	*E. coli*	environment	10
S15	*K. pneumoniae*	environment	5
S16	*P. aeruginosa*	environment	5
S17	*C. albicans*	human	5

## 4 Conclusions and Outlook

This study describes the biological generation of Ag-NPs in aqueous media via a simple, cost-effective, and eco-friendly bioreduction strategy. The Ag-NPs were synthesized from the AEPC extract, which contains different phytochemicals that act as reducing and stabilizing agents. The as-prepared Ag-NPs’ chemical structure and morphology were demonstrated using different analytical techniques, including UV–vis, FTIR, XRD, ζ-potential, TGA, SEM, EDX, and TEM. The characterization results indicate that the prepared Ag-NPs have a negative surface and are capped with various phytochemicals from the AEPC extract. The particle size distribution of the Ag-NPs ranges from 1 to 40 nm. The prepared Ag-NPs were first applied as a catalyst to reduce RhB dye in the presence of NaBH_4_ as a reducing agent. The results showed that 96% catalytic reduction can be accomplished after 32 min by using 20 μL of 0.05% Ag-NPs aqueous suspension in 100 μL of 1 mM RhB solution, 2 mL of DI water, and 1 mL of 10 mM NaBH_4_ solution. The kinetics of the catalytic reduction reaction was explained through the theoretical zero-order kinetic module since the experimental data results fitted well with the module and gave a rate constant of 0.059 mol L^–1^ min^–1^ and an *R*^2^ value greater than 0.98 for RhB dye. Furthermore, the prepared Ag-NPs were further utilized as antimicrobial agents against different Gram-positive, Gram-negative, and fungal microorganisms, and the results revealed high antimicrobial activity. Thus, the Ag-NPs prepared from the AEPC extract are promising catalysts for the remediation of contaminated water from RhB dye and microorganisms due to their easy synthetic strategy, unique structure, cost-effectiveness, environmentally friendly starting materials, high catalytic radiation efficiency, and rapid contact time.

## Supporting Information Available

Recyclability check of Ag-NPs (Scheme S1); GC-MS data of the phytochemicals in the aqueous AEPC extract in terms of their retention times (R. time) in minutes, % peak area, similarity index (S.I), and molecular weight in (g/mol) (Table S1); UV–vis spectra of the Ag-NPs synthesis as time progresses at room temperature and the nanoparticle’s color changes (Figure S1); parameters to calculate Ag-NPs sizes from Scherrer equation (Table S2); EDS elemental composition of the Ag-NPs (Table S3); EDS spectrum of the Ag-NPs from the AEPC extract (Figure S2); SEM and TEM images of green-synthesized Ag-NPs for RhB dye degradation of up to three cycles of applications (Figure S3); comparison of the zero-order rate constants of RhB catalytic reduction using the Ag-NPs from AEPC extract with the Ag-NPs in the literature (Table S4); and comparison between previous antimicrobial studies using Ag-nanoparticles with our study (Table S5) (PDF)
