# Supplementary material for: Green Synthesis of Silver Nanoparticles Using Acacia ehrenbergiana Plant Cortex Extract for Efficient Removal of Rhodamine B Cationic Dye from Wastewater and the Evaluation of Antimicrobial Activity
Source: ACS Omega. 2023 May 15;8(21):18901–14. doi: 10.1021/acsomega.3c01292 (PMC10233848; doi:10.1021/acsomega.3c01292)
Supplement: Supplementary file 1 — ao3c01292_si_001.pdf [file ao3c01292_si_001.pdf]

Green Synthesis of Silver Nanoparticles Using *Acacia Ehrenbergiana* Plant Cortex Extract for Efficient Removal of Rhodamine B Cationic Dye from Wastewater and the Evaluation of Antimicrobial Activity

Waleed M. Alamier[1]*, Mohammed D. Y. Oteef [1], Ayyob M. Bakry[1]*, Nazim Hasan[1], Khatib Sayeed Ismail[2], and Fathi S. Awad[3]*

[1] Department of Chemistry, Faculty of Science, Jazan University, Jazan 45142, Saudi Arabia.
[2] Department of Biology, Faculty of Science, Jazan University, Jazan 45142, Saudi Arabia.
[3] Chemistry Department, Faculty of Science, Mansoura University, Mansoura 35516, Egypt.

Supporting Information

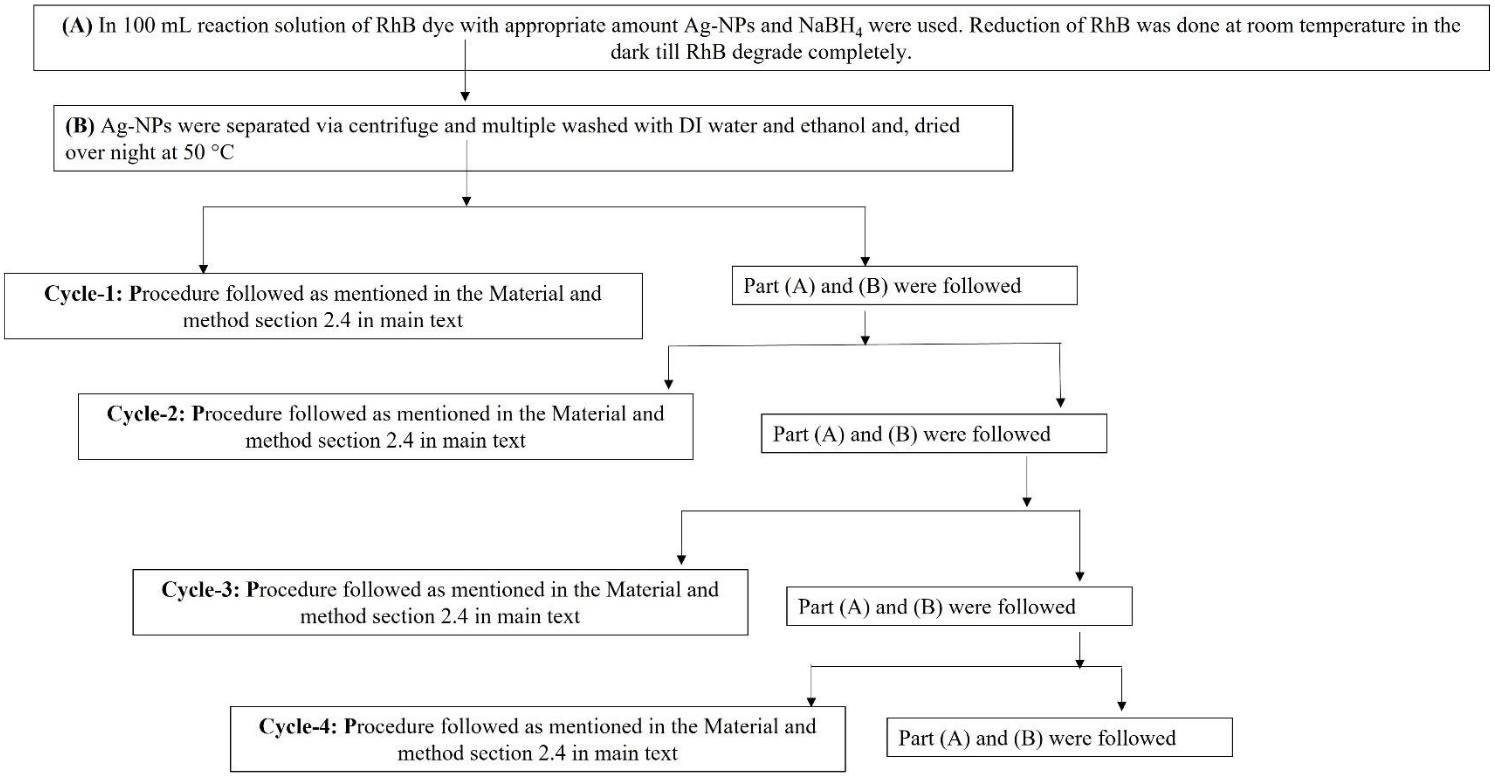

Scheme S1: Recyclability procedure of the AgNPs

Table S1: GC-MS data of the phytochemicals in the aqueous AEPC extract in terms of their retention times (R. Time) in minutes, % Peak area, Similarity Index (S.I) and Molecular weight in (g/mol.).

Peak No.	R. Time (min)	Peak area (%)	S.I.	Name of Phytochemical	Molecular weight (g/mol.)
1	3.089	0.59	93	Glycerin	92
2	4.382	0.08	93	Methyl valerate	116
3	6.380	0.09	93	2(5H)-Furanone	84
4	6.569	0.23	90	1,2-Cyclopentanedione	98
5	11.037	2.52	98	o-Guaiacol	124
6	14.070	0.35	95	n-Dodecane	170
7	16.409	0.73	94	Hydroquinone	110
8	17.347	1.04	88	4-Hydroxy-3-methylacetophenone	150
9	18.343	1.19	95	Syringol	154
10	19.655	0.49	88	Vanillin	152
11	20.903	1.03	85	Raspberry ketone	164
12	22.902	0.27	84	Guaiacylacetone	180
13	23.695	0.34	81	3,5-Dimethoxyacetophenone	180
14	24.711	1.24	93	3,4,5-Trimethoxyphenol	184
15	25.607	0.98	85	Homovanillic acid	182
16	26.767	71.48	82	3-O-Methyl-D-glucose	194
17	27.585	3.39	91	Coniferol	180
18	31.284	1.19	96	Methyl palmitate	270
19	35.049	1.32	97	Methyl stearate	298
20	36.026	0.98	92	Palmitamide	255
21	37.302	1.44	86	1,8-Diazacyclotetradecane-2,7-dione	226
22	39.120	3.92	93	Oleamide	281
23	39.526	0.49	90	Stearamide	283
24	41.450	3.04	92	2-Monopalmitin	330
25	44.524	1.58	94	1-Monostearin	358

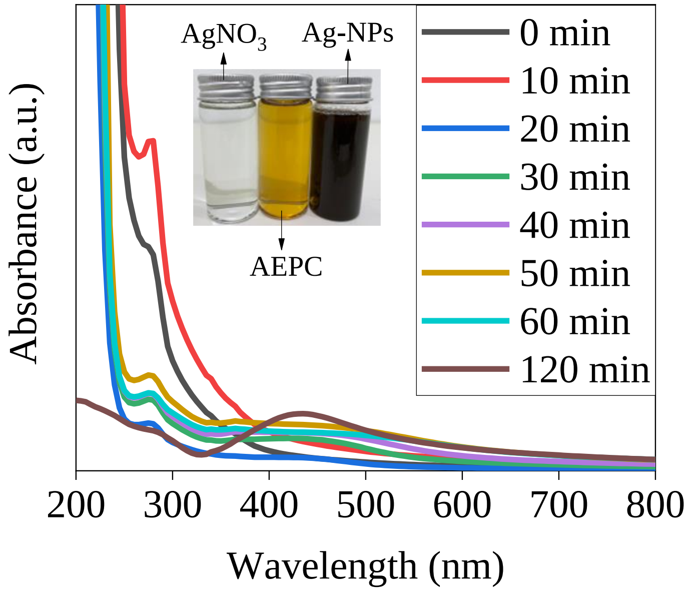

Figure S1. The UV–Vis spectra of the Ag-NPs synthesis as time progress at room temperature and the nanoparticle's color changes.

Scherrer Equation

$$D = 0.94\ \lambda / \beta\ \mathrm{Cos} \qquad \text{(S1)}$$

Where: β = Line broadening in radians, θ = Bragg angle, λ = X-Ray wavelength, and D = Average Crystallite size.

Table S2: parameters to calculate Ag-NPs sizes from Scherrer Equation

λ (nm)	2θ (°)	θ (°)	β	D (nm)
0.15458	38.3	19.15	0.8	10.98
0.15458	44.3	22.15	0.5	17.92
0.15458	64.6	32.3	0.5	19.64
0.15458	77.4	38.7	0.5	21.28

Table S3: EDS Elemental Composition of the Ag-NPs

Element	Weight %	Atomic %	Net Int.
C K	19.74	49.18	70.17
O K	16.07	30.05	32.48
S K	0.79	0.73	16.32
Cl K	4.3	3.63	84.88
Ag L	59.11	16.4	544.28

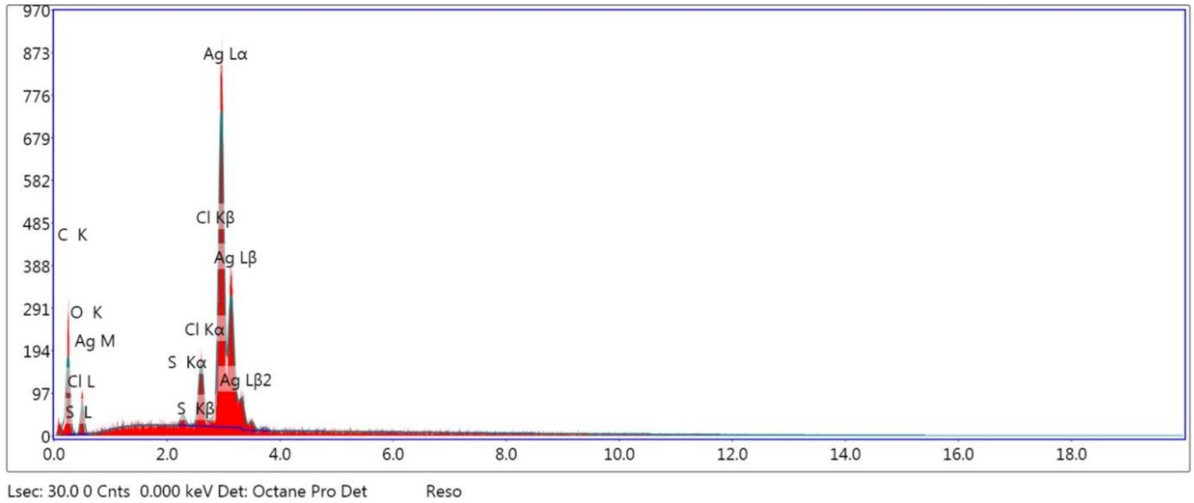

Figure S2. EDS spectrum of the green synthesized Ag-NPs obtained from the AEPC aqueous extract.

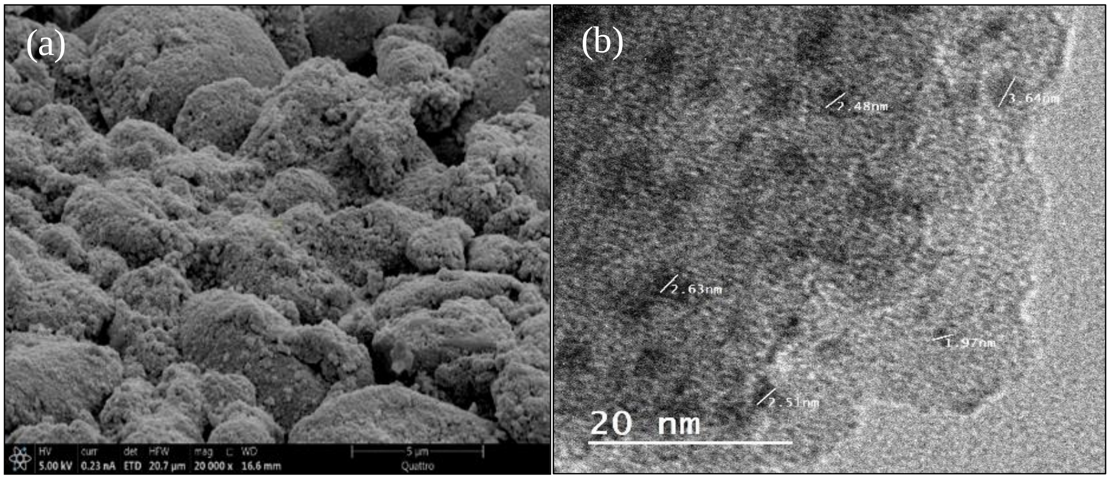

Figure S4. (a) SEM and (b) TEM images of green synthesized Ag-NPs for RhB dye degradation up to three cycles.

Table S4. A comparison of the zero-order rate constants of RhB catalytic reduction using the Ag-NPs from AEPC extract with Ag-NPs in the literature.

Catalyst	Biological system	Size (nm)	Dye	Rate constant (mol.L^{-1}.min^{-1})	Reference
Ag-NPs	Caralluma acutangula plant extract	2 – 6	Methelene Blue	0.0311	1
Ag-NPs	Caralluma acutangula plant extract	2 – 6	Cingo Red	0.0431	1
Ag-NPs	Acacia Ehrenbergiana plant cortex	1 – 40	Rohdamine B	0.0590	This work

Table S5: Comparison between previous antimicrobial studies using Ag-nanoparticles with our study.

No	Common bacteria studied	Plant used for NP synthesis	Antibiotic sensitivity testing Method	Zone of Inhibition (mm)	MIC (µg/mL)	Susceptibility/ Resistance to the AgNPs	Agreement	Ref
1	*S. aureus*<br>*B. cereus*<br>*E. coli*<br>*P. aeruginosa*<br>*C. albicans*	*Cassia roxburghii*	Synergistic activity with Antibiotics	AgNP alone didn't show inhibitory action, showed synergistic action with Antibiotics	-	Susceptible with Antibiotics	Agree	2
2	*E. coli*	*Cestrum nocturnum*	Micro dilution	23	8	Susceptible	Agree	3
3	*S. aureus*<br>*E. coli*	*Azadirachta indica*	Disc diffusion	9<br>9	-<br>-	Susceptible<br>Susceptible	Agree	4
4	*E. coli*	*Capsicum frutescence*	Agar well diffusion	11.5	80	Susceptible	Agree	5
5	*S. aureus*<br>*E. coli*<br>*K. pneumoniae*	*Lippia nodiflora*	Disc diffusion	20.3±0.32<br>22.0±1.5<br>20.4±0.76	50<br>50<br>50	Susceptible<br>Susceptible<br>Susceptible	Agree	6
6	*E. coli*<br>*K. pneumoniae*<br>*P. aeruginosa*	*Salvia officinalis*	Disc diffusion	37.86±0.21<br>25.18±0.27<br>14.27±0.08	100<br>50<br>50	Susceptible<br>Susceptible<br>Susceptible	Agree	7
8	*E. coli*	*Microwave assisted*	OD growth curve	-	50	Susceptible	Agree	8
9	*S. aureus* (susceptible strain)<br>MRSA<br>*E. coli*	Sr & Ag loaded nanotubular structures	Zone of inhibition	17<br><br>19<br>18	40<br><br>40<br>40	Susceptible<br><br>Susceptible<br>Susceptible	Agree	9
10	*S. aureus*<br>*E. coli*<br>*P. aeruginosa*<br>C. albicans	*Rhus coriaria L.*	Well diffusion	10±0.30<br>-<br>14±0.5<br>12±0.2	-<br>-<br>-<br>-	Susceptible<br>Susceptible<br>Susceptible<br>Susceptible	Agree	10

11	*S. aureus*<br>*E. coli*<br>*P. aeruginosa*	*Semecarpus anacardium, Golchidion lanceolarium & Bridelia retusa*	Micro dilution	43.94±0.2<br>44.02±0.3<br>68.6±0.5<br>Above results with Golchidion plant extract	70<br>80<br>100	Susceptible<br>Susceptible<br>Susceptible	Agree	11
12	*E. coli*	*Rhazya stricta*	Disc diffusion	Not stated	50	Susceptible	Agree	12
13	*E. coli*	*Yeast extract*	Micro dilution	Not applicable	20	Susceptible	Agree	13
14	*S. aureus*<br>*E. coli*<br>*K. pneumoniae*<br>*P. aeruginosa*	*Microwave irradiation method – Serine as reducing agent*	Modified Disc diffusion	16<br>21<br>12<br>21	50<br>50<br>50<br>50	Susceptible<br>Susceptible<br>Susceptible<br>Susceptible	Agree	14
15	*S. aureus*<br>*E. coli*		Tube double dilution	Not applicable	2	Susceptible<br>Susceptible	Agree	15
16	*S. aureus* (susceptible strain)<br>MRSA (10 strains)<br>*E. coli*<br>*K. pneumoniae*<br>P. aeruginosa<br>*C. albicans*	*Acacia ehrenbergiana*	Well diffusion	11<br>9 – 11<br>9<br>10<br>13<br>31	5<br>5–25<br>10<br>5<br>5<br>5	Susceptible<br>Susceptible<br>Susceptible<br>Susceptible<br>Susceptible<br>Susceptible	-	This work

References:

(1) Alamier, W. M.; Hasan, N.; Ali, S. K.; Oteef, M. D. Y. Biosynthesis of Ag Nanoparticles Using Caralluma acutangula Extract and Its Catalytic Functionality towards Degradation of Hazardous Dye Pollutants. Crystals 2022, 12 (8), 1069.

(2) Moteriya, P.; Padalia, H.; Chanda, S. Characterization, synergistic antibacterial and free radical scavenging efficacy of silver nanoparticles synthesized using Cassia roxburghii leaf extract. Journal of Genetic Engineering and Biotechnology 2017, 15 (2), 505-513. DOI: https://doi.org/10.1016/j.jgeb.2017.06.010.

(3) Keshari, A. K.; Srivastava, R.; Singh, P.; Yadav, V. B.; Nath, G. Antioxidant and antibacterial activity of silver nanoparticles synthesized by Cestrum nocturnum. J Ayurveda Integr Med 2020, 11 (1), 37-44. DOI: 10.1016/j.jaim.2017.11.003 From NLM.

(4) Ahmed, S.; Ullah, S.; Ahmad, M.; Swami, B.; Ikram, S. Green synthesis of silver nanoparticles using Azadirachta indica aqueous leaf extract. Journal of radiation research and applied sciences 2015, 9. DOI: 10.1016/j.jrras.2015.06.006.

(5) Shankar, T.; Karthiga, P.; Swarnalatha, K.; Rajkumar, K. Green synthesis of silver nanoparticles using Capsicum frutescence and its intensified activity against E. coli. Resource-Efficient Technologies 2017, 3 (3), 303-308. DOI: https://doi.org/10.1016/j.reffit.2017.01.004.

(6) Sudha, A.; Jeyakanthan, J.; Srinivasan, P. Green synthesis of silver nanoparticles using Lippia nodiflora aerial extract and evaluation of their antioxidant, antibacterial and cytotoxic effects. Resource-Efficient Technologies 2017, 3 (4), 506-515. DOI: https://doi.org/10.1016/j.reffit.2017.07.002.

(7) Yassin, M. T.; Mostafa, A. A.; Al-Askar, A. A.; Al-Otibi, F. O. Synergistic Antibacterial Activity of Green Synthesized Silver Nanomaterials with Colistin Antibiotic against Multidrug-Resistant Bacterial Pathogens. In Crystals, 2022; Vol. 12.

(8) Hong, X.; Wen, J.; Xiong, X.; Hu, Y. Shape effect on the antibacterial activity of silver nanoparticles synthesized via a microwave-assisted method. Environ Sci Pollut Res Int 2016, 23 (5), 4489-4497. DOI: 10.1007/s11356-015-5668-z From NLM.

(9) Cheng, H.; Xiong, W.; Fang, Z.; Guan, H.; Wu, W.; Li, Y.; Zhang, Y.; Alvarez, M. M.; Gao, B.; Huo, K.; et al. Strontium (Sr) and silver (Ag) loaded nanotubular structures with combined osteoinductive and antimicrobial activities. Acta Biomater 2016, 31, 388-400. DOI: 10.1016/j.actbio.2015.11.046 From NLM.

(10) Gur, T. Green synthesis, characterizations of silver nanoparticles using sumac (Rhus coriaria L.) plant extract and their antimicrobial and DNA damage protective effects. Front Chem 2022, 10, 968280. DOI: 10.3389/fchem.2022.968280 From NLM.

(11) Mohanta, Y. K.; Biswas, K.; Jena, S. K.; Hashem, A.; Abd Allah, E. F.; Mohanta, T. K. Anti-biofilm and Antibacterial Activities of Silver Nanoparticles Synthesized by the Reducing Activity of Phytoconstituents Present in the Indian Medicinal Plants. Front Microbiol 2020, 11, 1143. DOI: 10.3389/fmicb.2020.01143 From NLM.

(12) Shehzad, A.; Qureshi, M.; Jabeen, S.; Ahmad, R.; Alabdalall, A. H.; Aljafary, M. A.; Al-Suhaimi, E. Synthesis, characterization and antibacterial activity of silver nanoparticles using Rhazya stricta. PeerJ 2018, 6, e6086. DOI: 10.7717/peerj.6086 From NLM.

(13) Shu, M.; He, F.; Li, Z.; Zhu, X.; Ma, Y.; Zhou, Z.; Yang, Z.; Gao, F.; Zeng, M. Biosynthesis and Antibacterial Activity of Silver Nanoparticles Using Yeast Extract as Reducing and Capping Agents. Nanoscale Research Letters 2020, 15 (1), 14. DOI: 10.1186/s11671-019-3244-z.

(14) Jayaprakash, N.; Judith Vijaya, J.; John Kennedy, L.; Priadharsini, K.; Palani, P. Antibacterial activity of silver nanoparticles synthesized from serine. Mater Sci Eng C Mater Biol Appl 2015, 49, 316-322. DOI: 10.1016/j.msec.2015.01.012 From NLM.

(15) Yu, L.; Zhang, Y.; Zhang, B.; Liu, J. Enhanced antibacterial activity of silver nanoparticles/halloysite nanotubes/graphene nanocomposites with sandwich-like structure. Sci Rep 2014, 4, 4551. DOI: 10.1038/srep04551 From NLM.
